# An Awake Flexible Scope Intubation for a Patient With Trisomy 21, COVID-19, and Ludwig’s Angina

**DOI:** 10.7759/cureus.44370

**Published:** 2023-08-30

**Authors:** Marc Bozych, Emily Smith

**Affiliations:** 1 Anesthesiology, Nationwide Children’s Hospital, Columbus, USA; 2 Anesthesiology, Kaweah Health Medical Center, Visalia, USA

**Keywords:** ludwig's angina, covid-19 pneumonia, trisomy of 21, fiber-optic intubation, awake intubation

## Abstract

For patients with known or suspected atlantoaxial instability, awake flexible scope intubation is often an attractive option for safely securing the airway. Due to the challenges of consent and cooperation, patients with trisomy 21 are generally considered to be poor candidates for this technique. However, in rare instances, such as the case of this patient with co-existing Ludwig’s angina and COVID-19 pneumonia, the benefits of proceeding with an awake flexible scope intubation may outweigh the potential risks.

## Introduction

Patients with known or suspected cervical spine pathology must be handled delicately, with all airway manipulation performed from a neutral position to minimize the risk of causing spinal cord trauma. For many of these patients, an attractive option for securing the airway is awake flexible scope intubation, during which the patient can maintain their own position within an active range of motion and demonstrate intact neurologic function following passage of the breathing tube. Additionally, flexible scope intubation allows maintenance of spontaneous negative-pressure ventilation for a patient in whom airway collapse or obstruction may occur with muscle paralysis and positive-pressure ventilation, as may be the case for a patient with Ludwig’s angina. Furthermore, flexible scope intubation is now seen as the preferred first-line approach to securing the airway for these patients, supplanting the previous gold standard technique, a surgical airway [1-5].

Pneumonia in general (and COVID-19 pneumonia specifically) is not by itself an indication for awake intubation but will negatively impact a patient’s respiratory reserve and therefore provide yet another reason to consider keeping the patient breathing spontaneously during intubation. The most important factors to consider prior to attempting an awake flexible scope intubation are the patient’s ability to consent and cooperate. Due to the variable level of developmental delay associated with trisomy 21, these patients may not be able to provide consent (or assent) and/or may not be able to cooperate with the required steps of awake intubation; therefore, general anesthesia is usually required. This case report seeks to illustrate, for training purposes, an important example of when clinical practice may need to deviate from standard practice for the benefit of the patient [6].

## Case presentation

A 38-year-old female with trisomy 21 and diabetes mellitus presented to our hospital with facial and neck swelling, drooling, and intermittent shortness of breath eight days after testing positive for COVID-19 and failing antibiotic and steroid treatment prescribed by her family doctor. Due to her nonverbal status at baseline, history was obtained from her sister and primary caretaker.

A physical exam revealed tachycardia into the 110's, with vital signs otherwise within normal limits. She was breathing adequately from a sitting position but could not lie flat. She was additionally producing copious oral secretions, demonstrated trismus and oral thrush, and had submandibular swelling, induration, warmth, and tenderness.

**Figure 1:**
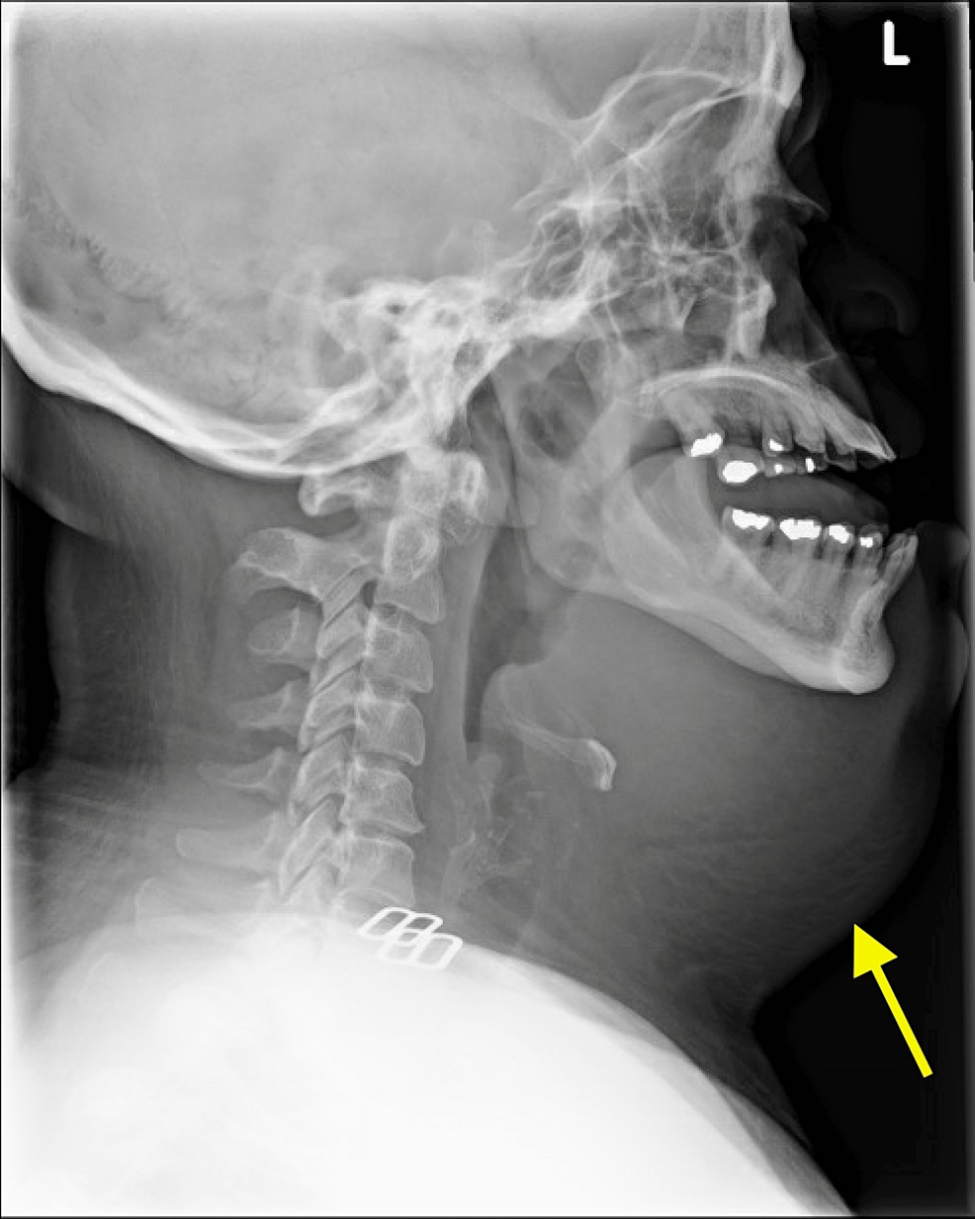
Lateral flat plate radiograph demonstrating submandibular soft tissue swelling (arrow)

Laboratory studies showed leukocytosis with left shift, respiratory acidosis, and elevated lactate, alkaline phosphatase, and C-reactive protein. A flat plate radiograph of the neck showed asymmetrical soft tissue swelling with narrowing near the epiglottis (Figure 1).

Airway equipment was brought to the patient’s room. Emergency department staff, two nurse anesthetists, an anesthesia technician, and a general surgeon were present to assist. Specific roles were assigned to all team members, and the plan for all maneuvers, up to and including tracheostomy, was discussed. American Society of Anesthesiologists (ASA) standard monitors were utilized throughout. Lidocaine 4% solution was administered via nebulizer for topical numbing of the upper airway. Glycopyrrolate 0.2 mg was then administered intravenously as an antisialagogue. A total of 105 mg of ketamine was given intravenously in small boluses during the procedure to keep the patient calm.

The oropharynx was first visualized via indirect laryngoscopy, which was tolerated well. The bronchoscope was then introduced using the video laryngoscope to assist with guidance and orientation, but despite suctioning and position changes, our view was frequently obscured by purulent fluid. Substantial swelling was noted in both the false vocal cords and surrounding tissues. Multiple attempts to pass the bronchoscope failed, with breaks taken intermittently to prevent oxygen desaturation below 89%, allow recovery from bradycardic episodes, and minimize psychological trauma. The video laryngoscope blade was then exchanged for a larger one, with immediate subsequent success in passing the bronchoscope through the vocal cords. A 6.5-mm tube was advanced into the trachea with moderate resistance appreciated at the cords. Proper positioning within the trachea was confirmed with colorimetric capnography and symmetric bilateral breath sounds. Subsequent migration of the tube into the mainstem bronchus resulted in oxygen desaturation, which was remedied by backing the tube out slightly, with appropriate tube positioning above the carina confirmed via chest radiograph.

A propofol drip was then started at 10 mcg/kg/min, with slow titration up to 40 mcg/kg/min. Rocuronium 50 mg was administered to prevent ventilator dyssynchrony, and fentanyl was added for additional sedation at 25 mcg/hr.

**Figure 2:**
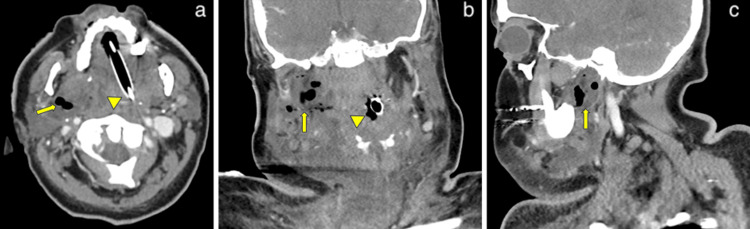
Contrast-enhanced computed tomography demonstrates submandibular edema and right parapharyngeal fluid and gas collection (arrows) at the C1-C2 levels in the transverse (a), frontal (b), and sagittal (c) planes, causing leftward deviation of the trachea (arrow heads).

The patient was then taken for maxillofacial computed tomography (CT), which demonstrated submandibular edema and parapharyngeal fluid and gas with associated narrowing of the oropharynx, consistent with Ludwig’s angina (Figure 2).

A chest CT revealed an abscess extending anteriorly to the thyroid and inferiorly to the first rib. Following 24 hours of intravenous antibiotics, the otolaryngologist performed an incision and drainage. Several days later, she was extubated under the supervision of the anesthesia team and eventually discharged to a skilled nursing facility.

## Discussion

This case report illustrates an incredibly rare combination of comorbidities. Our patient’s diabetes placed her at an increased risk of developing Ludwig’s angina [7], which has an incidence of 4%-8% within the realm of deep neck space infections [8, 9], but in our search of the scientific literature, we were unable to locate any previous cases describing the co-occurrence of Ludwig’s angina and COVID-19 pneumonia in a patient with trisomy 21. The significant swelling and deviation of the airway anatomy (including increased skin-to-trachea distance) would have made a surgical airway approach in this patient extremely challenging, if not outright futile. A flexible scope intubation therefore represented the most promising path toward successfully protecting her airway. The very precisely documented sequence of events for this patient’s care in the hospital made an accurate reconstruction of events possible.

One of the limitations of this case report has been the inability to directly obtain the patient’s perspective on the course of events leading to her hospitalization. Additionally, the patient’s follow-up care since discharge to the rehabilitation facility has not been well documented. Despite excellent home care provided to the patient by her sister, it is likely that the patient’s developmental delay and communication limitations played a role in the timeframe in which she presented to us for care.

## Conclusions

The practice of medicine represents both science and art and even though clinical guidelines exist to provide best practice recommendations based on consensus data, situations do occasionally arise involving multiple competing relative contraindications. When this occurs, a gestalt view of the circumstances must be taken and judgment calls made in order to proceed in the safest way possible. This patient’s very complicated constellation of medical problems required a nuanced approach to protect her from possible spinal cord trauma while addressing an impending respiratory collapse in the setting of challenging upper airway anatomy and pathology. Ordinarily, a nasopharyngeal flexible scope intubation may assist the clinician in bypassing edematous tissues in the mouth. However, a patient with trisomy 21 will tend to have a hypoplastic nasopharynx, which is why we chose to proceed with an oropharyngeal approach for this patient.
